# In-Situ Polymerization of High-Molecular Weight Nylon 66 Modified Clay Nanocomposites with Low Apparent Viscosity

**DOI:** 10.3390/polym11030510

**Published:** 2019-03-18

**Authors:** Xiaochao Duan, Yanpeng Wu, Zhao Chen, Tonghui Yang, Yongchang Cheng, Hao Yu, Tao Huang

**Affiliations:** State Key Lab for Modification of Chemical Fibers & Polymer Materials, College of Materials Science & Engineering, Donghua University, Shanghai, China No. 2999 North Renmin Road, Songjiang District, Shanghai 201620, China; Duanxc@mail.dhu.edu.cn (X.D.); wuyanpeng334@163.com (Y.W.); acczhao@163.com (Z.C.); thui_yang@163.com (T.Y.); chengyongchangniou@163.com (Y.C.)

**Keywords:** nylon 66/clay nanocomposites, shear viscosity or melt viscosity, in situ

## Abstract

High-molecular weight nylon 66/modified clay (Mclay) nanocomposites with a low apparent viscosity were prepared by in-situ polymerization and post solid-state polycondensation. Thermogravimetric analysis and X-ray diffraction patterns of the Mclay revealed that cetyltrimethyl ammonium bromide successfully inserted into the interlayers of the clay. Scanning electron microscope images of the cross sections showed that the Mclay was well-dispersed in the nylon 66 matrix. The effects of clay on the mechanical, rheological, and thermal properties of the nanocomposites were investigated using an Instron 5969 machine, a capillary rheometer, and a differential scanning calorimeter. The results indicated that the incorporation of a very small amount of Mclay considerably decreased the shear viscosity of the nanocomposites and increased the melt index, acting as a viscosity reducer. More importantly, the mechanical properties and spinnability of the nylon 66/Mclay nanocomposites were not affected by the viscosity reducer.

## 1. Introduction

Nylon 66 (PA66 or polyamide 66) is a versatile engineering plastic within the nylon family. It is widely used because of its light weight, good mechanical properties, high abrasion resistance, excellent chemical resistance, and relatively low cost [1,2,3,4,5]. However, many unmodified polymer materials cannot meet the increasingly demanding requirements of specific fields, and nylon 66 also has some limitations. Hence, nylon 66 composites have been developed in recent years to enhance the performance of nylon 66 and meet new demands. Nylon 66 modified with nanofillers has been a topic of research interest for the past 30 years.

Polyamide/layer silicate composites have attracted considerable attention since they were first reported by Okada, in 1987 [6]. Nylon 66/clay nanocomposites have also received considerable attention due to their superior properties, such as flame retardancy [7,8], high tensile strength [9], and high modulus [10]. Song et al. prepared nylon 66/clay nanocomposites via condensation polymerization, which showed enhanced thermal stability and flame-retardant properties compared with those features of pure PA66 [8]. Han et al. prepared nylon 66/montmorillonite nanocomposites via melt-compounding in a twin extruder, resulting in a significant enhancement of the mechanical strength and Young’s modulus of the resulting composites [11]. The above-mentioned clay effectively improved the flame retardancy and mechanical properties of nylon 66. However, the apparent viscosity of the nylon 66 composites increased, which could potentially cause problems for the subsequent processing performance of nylon 66 [12,13]. 

There is typically a trade-off between high molecular weight and good flowability, whereas poor flowability has a greater effect on the spinnability of polymer melt. Thus, there is a need to reduce the viscosity of polymers without reducing their strength. Paul et al. [1] prepared nylon 6 organoclay nanocomposites using a melt blending method. The resulting nanocomposites showed a lower apparent viscosity than that of pure PA6 and, although the yield strength of the nanocomposites increased, the elongation at break decreased [14]. Although some reports have suggested a low apparent viscosity of nylon 6 clay composites, there have been few reports on reducing the apparent viscosity of the melt of high-molecular weight nylon 66 without changing its mechanical properties. Therefore, reducing the viscosity of nylon 66 composites while maintaining their high mechanical properties remains a desirable target.

Herein, we report an effective approach to preparing high-molecular weight nylon 66/modified clay (Mclay) nanocomposites on the basis of an in-situ intercalative polymerization and post solid-state polycondensation. These results indicate that the clay enhanced the tensile strength of the nylon 66/Mclay nanocomposites. More importantly, the apparent viscosity of the nylon 66/Mclay nanocomposites was reduced, enabling easy processing.

## 2. Materials and Methods 

### 2.1. Materials

Clay powder (laponite RD) was obtained from Nanjing Baiyike New Material Technology Co., Ltd. (Nanjing, China). The PA66 salt was purchased from BASF SE (Shanghai, China). Cetyltrimethyl ammonium bromide (CTAB), AgCl, AgNO_3_ and concentrated sulfuric acid was purchased from Sinopharm Chemical Reagent Co., Ltd. (Shanghai, China). Deionized water was prepared in the laboratory. All the used reagents were received from commercial sources.

### 2.2. Preparation of Modification of Clay

Clay (1.5 g) was homogeneously dispersed in 200 mL of deionized water at 80 °C, with magnetic stirring for 20 min. A 43 g solution of CTAB (1 wt % in deionized water) was gradually added to the aqueous clay dispersion. This was followed by further vigorous magnetic stirring for 8 h. The treated clay was obtained as a white precipitate, which was repeatedly washed with hot deionized water to remove residual CTAB. The filtrate was titrated with 0.1 mol/mL AgNO_3_ until no precipitate of AgCl was formed, to ensure the complete removal of Cl^−^. The product was placed in a vacuum drying oven at 80 °C for 8 h. The dried product was ground to obtain the Mclay. 

### 2.3. Polymerization of Nanocomposites of Nylon 66 and Mclay

A typical procedure to prepare nylon 66/Mclay nanocomposites involving clay dosages of 0.1 wt% (PA66-Mclay-0.1) was as follows: Mclay (1.5 g) was added into a PA66 salt solution at a concentration of 60 wt % at 80 °C. The above mixtures were placed into a polymerization reactor (5 L) after mechanical stirring for 20 min. The air in the reactor was replaced by evacuation and nitrogen injection at least three times before heating up. The mixtures were then heated at 220 °C for 2 h at a high pressure of 1.7 MPa and then evacuated to atmospheric pressure, at 280 °C for 2 h, with steady stirring. Nylon 66 composites with different clay contents (PA66-Mclay-0.05, PA66-Mclay-0.075, PA66-Mclay-0.5) were prepared by the same technique. The pure PA66 was also prepared using the same technique without the Mclay. Chips of the PA66 and PA66/Mclay nanocomposites were placed in a vacuum drying oven at 200 °C for 4 h under a pressure of 100 Pa, to further increase the molecular weight.

### 2.4. Melting Spinning of Nanocomposites of Nylon 66 and Mclay

Pellets of nylon 66 or nylon 66/Mclay nanocomposites were melt-spun on a lab-scale melt spinner with a single-screw extruder (*D* = 25 mm, *L*/*D* = 28). The extruded fibers were collected as they exited the spinneret orifice. The take-up fibers were quenched with cross air and collected at 800 m/min. The main spinning parameters are summarized in Table 1.

### 2.5. Characterization

Thermo-gravimetric analysis (TGA) was used to characterize the intercalation of the Mclay, performed with a TG 209 F1 (Netzsch, Serb, Germany) under a nitrogen atmosphere at a flow rate of 20 mL/min. A 4 mg portion of the powder samples was heated from ambient temperature to 900 °C at a heating rate of 20 °C/min. Differential scanning calorimetric (DSC) testing was performed with a Netzsch 204 F1. The dried samples, under a nitrogen atmosphere, were first heated to 290 °C at a rate of 20 °C/min, and held at that temperature for 5 min to completely remove the previous thermal history, before cooling to room temperature at a rate of 10 °C/min. Finally, the sample was heated to 290 °C again at a rate of 10 °C/min. The cross-section morphologies of the of nylon 66/Mclay nanocomposites were observed with a field emission scanning electron microscope (FESEM, S-4800, Hitachi, Toyko, Japan). The X-ray diffraction (XRD) analyses were performed using HD-D/max-2550VB+/PC X-ray diffraction (Akishima, Japan). The samples for mechanical property tests were prepared by injection molding. Tensile strength and elongation at break were measured according to GB/T 1040.2-2006, and the strain rate was 20 mm/min. Mechanical testing was performed using an electronic universal testing machine (Instron 5969). Rheological measurements were performed with a capillary viscometer, RH2000 (Malvern, UK), at 290 °C. The capillary was 1 mm in diameter and had a length-to-diameter ratio of 16.

The viscosity number (VN) was obtained as follows: First, clay was removed from the composites by centrifuging the formic acid solution of the nanocomposites. The PA66 was then precipitated from the supernatant liquor with deionized water, repeatedly washed by ultrasonication in deionized water until the washings became neutral, and then dried at 85 °C in vacuum overnight. The viscosity number (VN) reflects the molecular weight of PA66 and its nanocomposites. It was measured with an Ubbelohde viscometer at 25 °C by dissolving the samples in 96% concentrated sulfuric acid with a concentration of 5 mg/mL, according to ISO 307: 2007. The intrinsic viscosity and end-group (–NH_2_, –COOH) content were measured with an Ubbelohde viscometer at 25 °C by dissolving the samples in 96% concentrated sulfuric acid with a concentration of 5 mg/mL and a titration according to Burke et al. [15].

## 3. Results and Discussion

### 3.1. Characterizations of the Modified Clay

**Figure 1:**
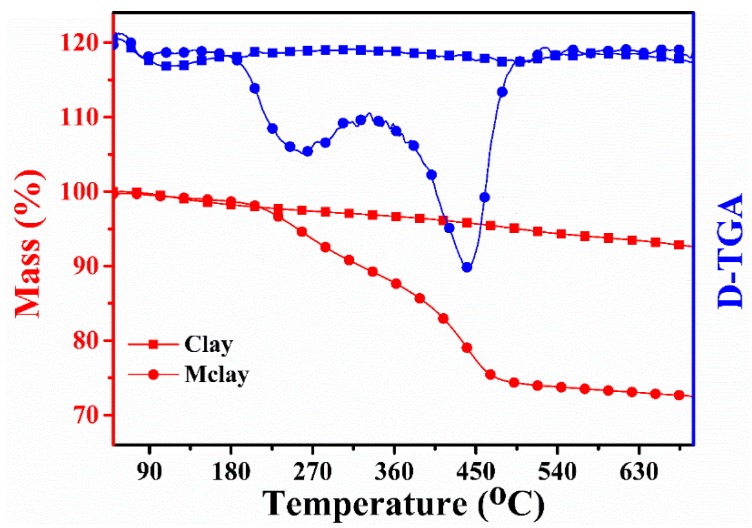
The thermo-gravimetric analysis (TGA) curves of clay and modified clay (Mclay).

The thermal stability data of the clay and Mclay are shown in Figure 1. The thermal stability of the clay was much better than that of the Mclay. The clay had a mass loss of up to 170 °C from the evaporation of adsorbed water. A further small mass loss was observed over the region of 350–570 °C, owing to the removal of the interlayer water. Compared with the clay, the Mclay showed a sharp mass loss of adsorbed water, observed over the temperature range of 180–530 °C, which was attributed to the decomposition of CTAB in the interlayer of the clay. 

**Figure 2:**
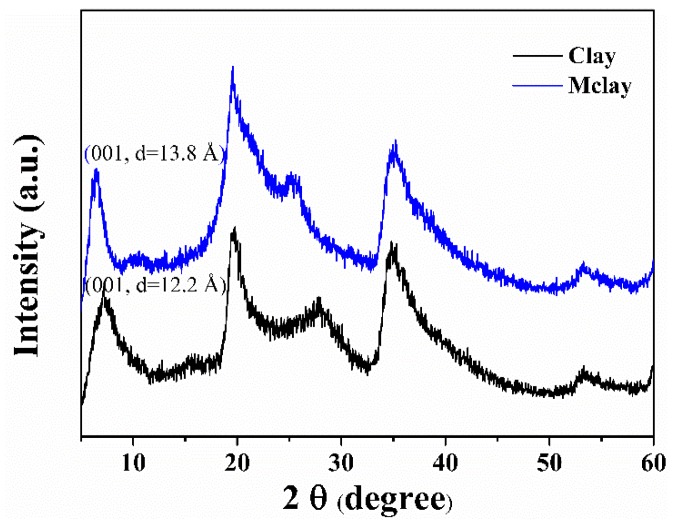
The XRD patterns of clay and Mclay.

The XRD patterns of the clay and Mclay are shown in Figure 2. Diffraction peaks at 7.2° (*d* = 12.2 Å) and 6.4° (*d* = 13.8 Å) were detected for clay and Mclay, respectively, and were assigned to the (001) crystal plane or basal spacing of the clay, indicating that CTAB intercalated within the clay interlayer and increased the basal spacing [16]. Thus, a monomer intercalated into the clay interlayer and assisted in the exfoliation of the clay during the polymerization process. Additional peaks were present at 19.4°, 27.5°, 33.7°, and 60.4°, corresponding to (100), (005), (110), and (330) crystal planes, respectively. Pure clay and Mclay exhibited similar patterns, except for the (005) crystal planes.

### 3.2. Characterizations and Properties of Nylon 66 and Nylon 66/Mclay Nanocomposites

**Figure 3:**
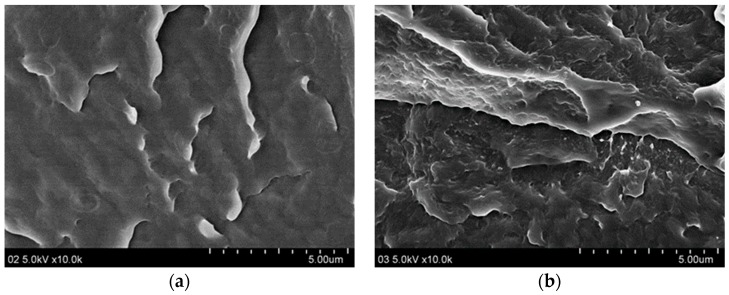
The SEM photographs of the cross section of PA66/Mclay nanocomposites: (**a**) PA66; (**b**) PA66-Mclay-0.1.

The cross-sectional image of the nylon 66/Mclay in Figure 3 shows a smooth fractured surface and no aggregations of Mclay sheets, indicating that the Mclay particles were well-dispersed in the PA66/Mclay composites. 

**Figure 4:**
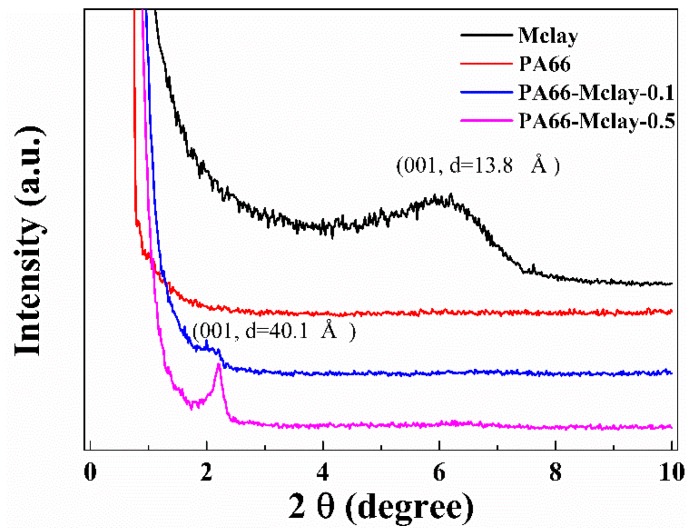
Small-angle XRD spectra of Mclay, nylon 66, and nanocomposites

Small-angle X-ray diffraction patterns for the Mclay powder, nylon 66, and the nanocomposites are shown in Figure 4. A diffraction peak at 2θ = 6.4° (*d* = 13.8 Å) in the Mclay, which is a reflection from the clay basal spacing, disappeared in the nanocomposites, and a new diffraction peak appeared at approximately 2θ = 2.2° (*d* = 44.1 Å). The new peak was likely generated from a widening of the 001 crystal plane, with the chain of nylon 66 widening the clay interlayer, but not resulting in complete exfoliation. This form of clay likely decreased the shear melt viscosity of the nanocomposites. 

**Figure 5:**
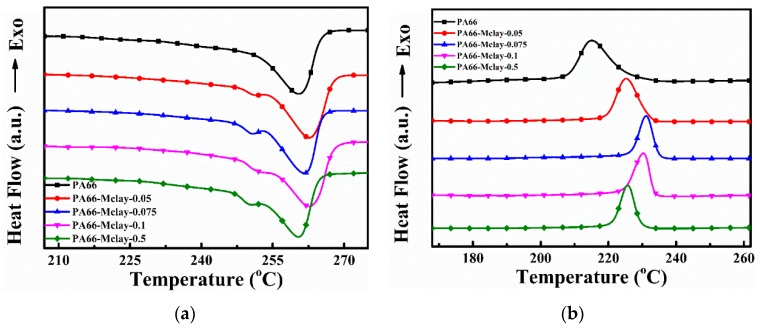
The DSC characterization of PA66 and PA66/Mclay nanocomposites: (**a**) DSC heating curve; (**b**) DSC cooling curve.

**Figure 6:**
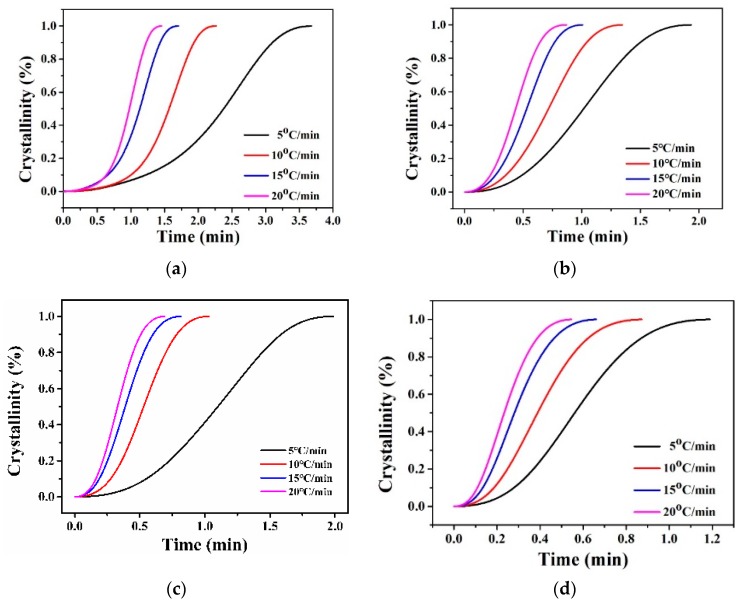

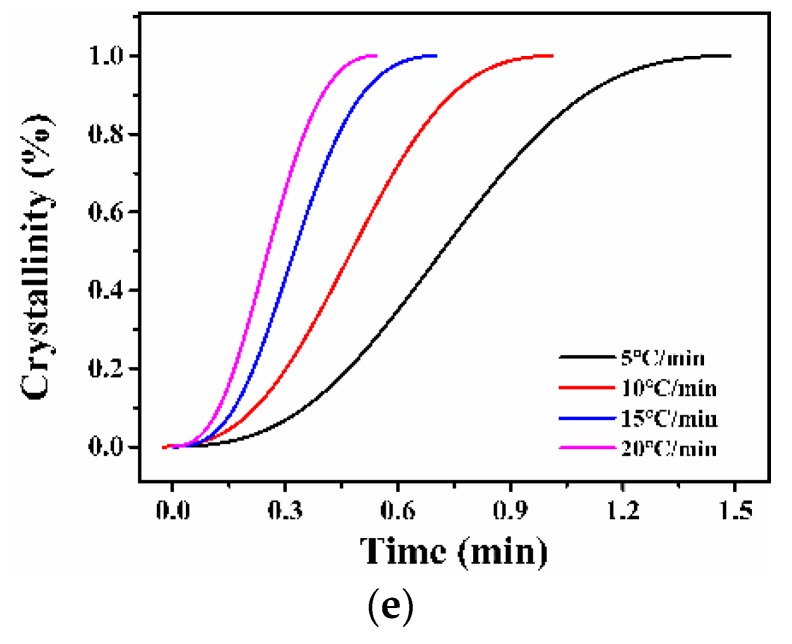
Relative crystallization time curves: (**a**) PA66, (**b**) PA66-Mclay-0.05, (**c**) PA66-Mclay-0.075, (**d**) PA66-Mclay-0.1, (**e**) PA66-Mclay-0.5.

The melting endotherm graphs of neat nylon 66 and nylon 66/clay nanocomposites during the second heating cycle are shown in Figure 5, and Table 2 lists the DSC data. The melting peaks fluctuated slightly at around 261 °C. However, the pure nylon 66 in Figure 5a exhibited only one melting peak [17] and the nanocomposites exhibited two melting peaks, which was similar to our previous reports [18]. The main melting temperature (*T*_m__I_) at 260 °C is attributed to a Form_I_, or α-type peak [3]. The second melting temperature (*T*_m__II_) at 252 °C was attributed to a Form_II_, or γ-type peak. These results indicate that introducing clay into PA66 increased the amount of γ-form crystals and hindered the formation of perfect α nylon crystals during the heating cycle of the DSC measurements [19]. The crystallization exotherm curves of the samples during the cooling cycle are shown in Figure 5b. Pure PA66 had a wide crystallization peak, with a crystallization temperature (*T*_c_) peak at 215.0 °C. The nanocomposites with Mclay had much higher *T*_c_ values, which were approximately 15 and 10 °C higher than those of the pure polymer, respectively. The crystallization peaks of the nanocomposites were also narrower. This result suggests that the clay acted as a nucleation agent and increased the crystallization rate of the nanocomposites, which was further confirmed by the non-isothermal crystallization analysis in Figure 6 and Table 3. For example, at the same cooling rate of 10 °C/min, the half crystallization times of PA66, PA66-Mclay-0.05, PA66-Mclay-0.075, PA66-Mclay-0.1, and PA66-Mclay-0.5 were 1.57, 0.72, 0.53, 0.39, and 0.48 min, respectively. The addition of Mclay greatly increased the crystallization rate of the nylon 66/Mclay nanocomposites. Moreover, the crystallization temperature increased until the Mclay content was 0.075, and then it decreased. The relative crystallinity also increased and then decreased with the increases of the Mclay content. This phenomenon will be explained later.

**Figure 7:**
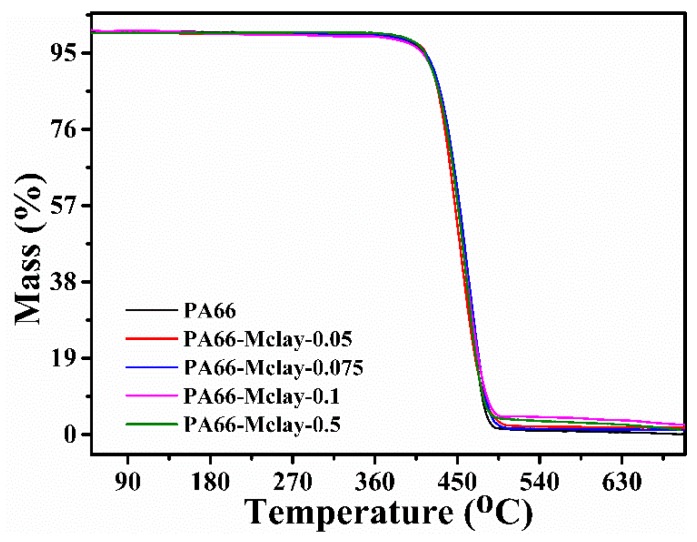
The TGA curves of PA66 and PA66/Mclay nanocomposites.

The evidence for the thermal stability of PA66 and PA66/Mclay nanocomposites was obtained through TGA (Figure 7). There were no significant differences between the TGA curves of the PA66 and PA66/Mclay nanocomposites, indicating that the addition of Mclay did not affect the thermal decomposition temperature of the PA66/Mclay nanocomposites. The carbon residue of the PA66/Mclay nanocomposites slightly increased with the increase of the Mclay loadings. These results indicate that the Mclay may promote the generation of residual carbon.

**Figure 8:**
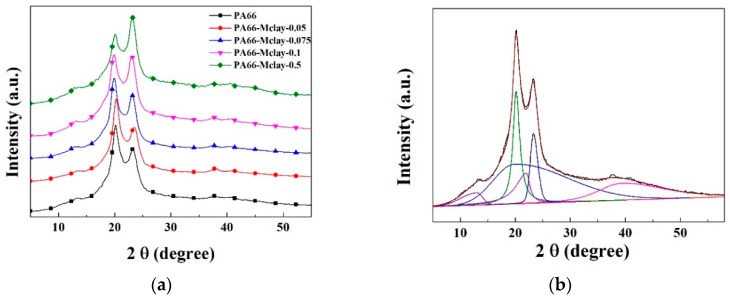

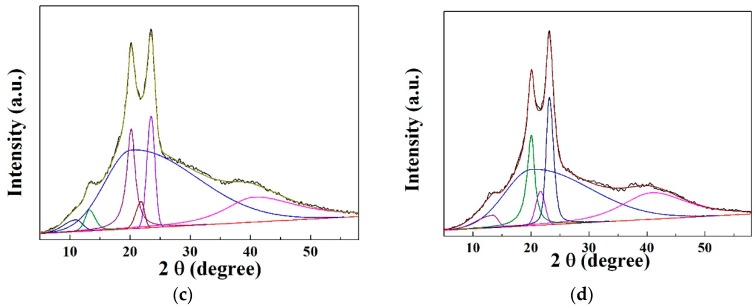
(**a**) The XRD pattern of nylon 66 and nylon 66/clay nanocomposites; (**b**) peak differentiation imitating of PA66; (**c**) peak differentiation imitating of PA66-Mclay-0.1; (**d**) peak differentiation imitating of PA66-Mclay-0.5.

Figure 8 and Table 4 shows the XRD patterns and detailed data of the neat nylon 66 and nylon 66/Mclay nanocomposites. The positions of the diffraction peaks of the neat nylon 66 nanocomposites at 20.1° and 23.3°, respectively, are reflections of the α1 and α2 form of the nylon 66 crystals [20], which did not change markedly, although the intensities were slightly different. The intensity of the α1 crystal decreased, and the α2 crystal increased, as the clay content increased. The α1 peak in the XRD pattern of nylon 66 was attributed to the distance between the hydrogen-bonded chains, reflecting the diffraction of the hydrogen-bonded sheets, whereas the α2 peak resulted from the separation of the hydrogen-bonded sheets. The sharp decrease in the intensity of the α1 peak in the nylon 66/Mclay nanocomposites indicates that the addition of clay disrupted the perfect arrangement of the hydrogen-bonded sheets in the α phase. As mentioned above, the crystallization temperature and the relative crystallinity from the DSC data increased and then decreased with the increase of Mclay content. The most likely reason for this outcome was the simultaneous actions of heterogeneous nucleation and the destruction of the perfect arrangement of the hydrogen-bonded sheets. Heterogeneous nucleation increases relative crystallinity and the crystallization temperature. At the same time, the relative crystallinity and the crystallization temperature are proportional to the density of the hydrogen bonds for a polyamide. Heterogeneous nucleation dominated the increase of the crystallization rate with a low Mclay content. As the content of Mclay increased, the destruction of the hydrogen bond also increased, causing the relative crystallinity and the crystallization temperature to decrease.

The viscosity number (VN) and intrinsic viscosity of bare nylon 66, and nylon 66 separated from nylon 66/Mclay nanocomposites, were determined by the Ubbelohde viscometer method. The VN and intrinsic viscosity of nylon 66 increased when clay was introduced into the nylon matrix, indicating that the molecular weight of nylon 66 increased as the content of the clay increased, as shown in Table 5. However, the melt index (MI) of the nanocomposite increased by 117.5% and 24.7% when the clay contents were 0.1 wt % and 0.5 wt %, respectively, despite the high molecular weight of the nanocomposites. This result indicates that the clay played an effective role in reducing the viscosity of nylon 66. 

**Figure 9:**
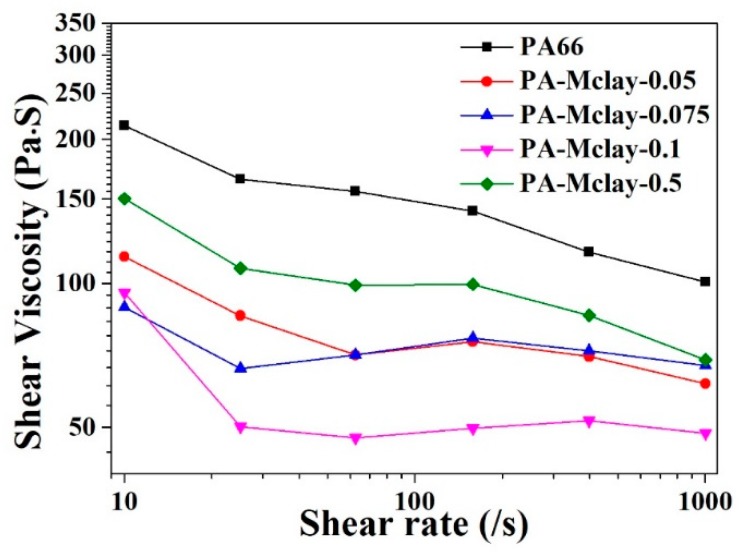
Melt viscosity and shear rate relationship at 290 °C.

The shear viscosity behaviors of nylon 66 and the various composites at high shear rates, measured by capillary rheometry, are shown in Figure 9. The nanocomposites exhibited a similar shear-thinning behavior to that of bare nylon 66. Notably, the absolute value of the melt viscosity of the clay nanocomposite was markedly lower than that of neat nylon 66, which is in accordance with the MI measurement. The low viscosity suggests that the nanocomposite would have good melt processability over a wide range of practical processing conditions, such as extrusion and injection molding. One possible mechanism which may have acted to reduce the melt viscosity in the nanocomposites was slippage between the nylon 66 matrix and the exfoliated clay platelets under high shear flow. When the shear rate was between 100–1000 s^−1^, the degree of viscosity reduction of the nanocomposites was less than that of pure nylon 66, which showed a more stable shear viscosity with an increased shear rate. This result is useful for improving the processing properties of nanocomposites, particularly during spinning processes. 

To study the effects of Mclay on the mechanical properties of nanocomposites, the properties of nanocomposites are shown in Table 6. The properties of neat nylon 66 are also shown in this table for comparison. The tensile properties of the Mclay composites were notably higher than those of the neat nylon 66, for nanocomposites. The Mclay acts as a viscosity-reducing agent to generate nanocomposites with low melt viscosity and good mechanical properties. 

### 3.3. Characterization and Properties of Nylon 66 and Nylon 66/Mclay Fibers

**Figure 10:**
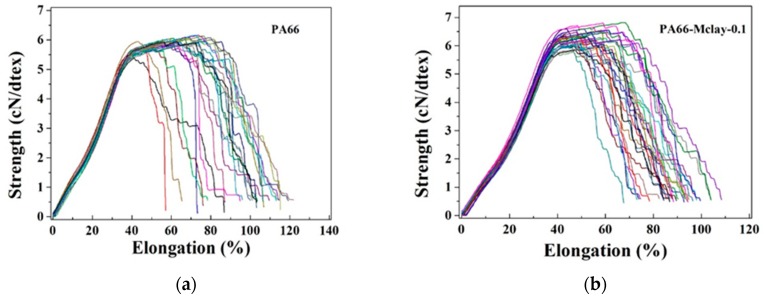
The tensile curve of nylon 66 and nylon 66/Mclay fibers: (**a**) PA66; (**b**) PA66-Mclay-0.1.

PA66 and PA66-Mclay-0.1 fibers were prepared by melt spinning, which indicated that the spinnability of PA66 was not affected by the viscosity reducer. Figure 10 and Table 7 show the mechanical properties of PA66 and PA66-Mclay-0.1 fibers. It is obvious that the tensile strength and modulus of the PA66-Mclay-0.1 fibers were greater than that of the pure PA66 fiber. This indicates that the Mclay improved the processing properties by reducing the viscosity of PA66 and simultaneously acted as a reinforcing agent to improve the mechanical properties of the PA66 fibers.

## 4. Conclusions

We prepared nylon 66/Mclay nanocomposites by in-situ polymerization of PA66 salt and modified the clay with CTAB. The XRD spectra showed that the CTAB successfully broadened the basal spacing of the Mclay, inducing the PA66 salt to enter into the interlayers of the clay much more easily, thereby promoting a homogeneous dispersion of Mclay in the nylon 66 matrix. The index and capillary rheometry results indicated that the Mclay was the key factor that decreased the viscosity and improved the post-processing performance of the nylon 66 melt. Furthermore, the mechanical properties of nylon 66/Mclay fibers were also improved, offering great promise for wider applications of PA66 materials and a new approach to producing high-molecular weight nylon 66 with low viscosity.

## Figures and Tables

**Table 1:** Process parameters of melt spinning.

Head pressure of extruder (MPa)	50
Number of spinneret orifice	36
Diameter of spinneret orifice (mm)	0.3
*L/D* of spinneret orifice	0.25
Barrel temperatures of extruder (°C)	285, 305, 300, 300
Temperature of spinneret (°C)	298
Flow rate in volume, *Qv* (cm^3^/min)	50
Temperature of cross air, *T* (°C)	25
Take-up velocity, *VL* (m/min)	800
Distance from spinneret to the first roller (m)	4.5

**Table 2:** Melting point, crystallinity, and crystallization temperatures of PA66 and PA66/Mclay samples from DSC data.

Sample	PA66	PA66-Mclay-0.05	PA66-Mclay-0.075	PA66-Mclay-0.1	PA66-Mclay-0.5
*T*_m__I_ (°C)	260.5	262.7	261.6	262.6	260.5
*T*_m__II_ (°C)	--	251.3	250.6	251.9	250.4
*X*_c_ (%)	20.87	21.24	22.15	24.69	21.26
*T*_c_ (°C)	215.0	225.3	231.1	230.2	225.7

**Table 3:** Semi-crystallization times at different cooling rates of PA66 and PA66/Mclay from DSC data.

		Sample	PA66	PA66-Mclay-0.05	PA66-Mclay-0.075	PA66-Mclay-0.1	PA66-Mclay-0.5
	T_1/2_ (min)						
Rate (°C/min)							
5			2.38	1.02	1.10	0.56	0.72
10			1.57	0.72	0.53	0.39	0.48
15			1.13	0.53	0.39	0.28	0.32
20			0.98	0.44	0.33	0.23	0.25

**Table 4:** The α peak, γ peak, and degree of crystallinity of PA66 and PA66/Mclay samples from XRD data through Jade software.

Sample	PA66	PA66-Mclay-0.05	PA66-Mclay-0.075	PA66-Mclay-0.1	PA66-Mclay-0.5
α_1_ (^o^)	20.1	20.2	19.9	19.8	20.1
α_2_ (^o^)	23.3	23.5	23.1	23.0	23.1
γ (^o^)	22.0	21.8	21.6	21.9	21.7
X_c_ (%)	33.9	34.2	34.7	33.8	25.5

**Table 5:** Viscosity number (VN), Relative viscosity (RV), [η], *M_v_*_,_ endgroup content, *M*_n_, and melt index (MI) of the PA66 and PA66/Mclay.

Sample	PA66	PA66-Mclay-0.05	PA66-Mclay-0.075	PA66-Mclay-0.1	PA66-Mclay-0.5
VN (ml/g)	184.3	183.2	185.6	189.6	186.7
RV (JIS)	3.35	3.33	3.37	3.42	3.38
[η] (dL/g)	1.12	1.11	1.12	1.13	1.13
*M_v_* ^a^	28,840	28,487	28,840	29,241	29,241
Endgroup content (mmol/kg)	81.02	80.44	79.77	79.35	79.55
*M*_n_	24,684	24,862	25,073	25,204	25,143
MI (g/10 min)	3.72 ± 0.27	5.75 ± 0.31	6.05 ± 0.41	8.09 ± 0.53	4.64 ± 0.14

^a^ [η] = *K*[*M*_v_] ^α^, *K* = 1.15 × 10^−3^, α = 0.67 [15].

**Table 6:** Tensile strength, modulus, and elongation at break of PA66 and PA66/Mclay.

Sample	Tensile Strength (MPa)	Tensile Modulus (MPa)	Elongation at Break (%)
PA66	79.5 ± 1.24%	515.4 ± 2.43%	434.2 ± 6.73%
PA66-Mclay-0.1	86.1 ± 0.87%	517.8 ± 2.78%	312.8 ± 7.18%
PA66-Mclay-0.5	88.9 ± 0.92%	519.5 ± 2.46%	642.5 ± 7.09%

**Table 7:** Fiber denier, orientation, and mechanical properties of fibers of nylon 66 and nylon 66/Mclay.

No.	Denier	Fiber Orientation	Tensile Strength (cN/dtex)	Elongation at Break(%)	Modulus (cN/dtex)
PA66	7828.2	0.74	5.9 ± 3.2%	63 ± 16.8%	9.6
PA66-Mclay-0.1	7493.4	0.74	6.3 ± 4.1%	51.5 ± 14.2%	13.1
